# Multiomics profiling of bone in the *Dpp3-*deficient mice unveils altered protein and lipid composition

**DOI:** 10.1007/s40618-026-02949-6

**Published:** 2026-08-03

**Authors:** Emilia Bevacqua, Federica Violi, Valentina Granata, Fabio Bedin, Mariarosaria Santillo, Ciro Menale, Gherardo Mazziotti, Cristina Sobacchi

**Affiliations:** 1 National Research Council, Institute for Genetic and Biomedical Research, Milan Unit, Milan, Italy; 2 Laboratory of Translational Endocrinology and Metabolism, Humanitas Research Hospital IRCCS, Bone Metabolism Unit, Rozzano, MI Italy; 3 Cellular and Humoral Innate Immunity Lab, Humanitas Research Hospital IRCCS, Rozzano, MI Italy; 4 San Raffaele Telethon Institute for Gene Therapy (SR-Tiget), Segrate, MI Italy; 5 Department of Clinical Medicine and Surgery, University of Naples “Federico II”, Naples, Italy; 6 Department of Biomedical Sciences, Humanitas University, Pieve Emanuele, MI Italy; 7 Metabolic Bone Diseases and Osteoporosis Section, Endocrinology, Diabetology and Andrology Unit, Humanitas Research Hospital IRCCS, Rozzano, MI Italy

**Keywords:** DPP3, Osteoporosis, Proteomics, Lipidomics, Mouse model

## Abstract

**Purpose:**

Bone is a unique specialized connective tissue comprising inorganic and organic components which continuously undergoes remodeling through the activity of anabolic and catabolic pathways. Peptidases are enzymes cleaving peptide bonds in a broad range of substrates and hence involved in many pathophysiological mechanisms. We previously reported that lack of Dpp3 in the mouse model results in bone loss and here we aimed to establish whether it also affected bone matrix composition.

**Methods:**

We performed proteomic and lipidomics analysis of the flushed bone of 6-month-old wild type (WT) and *Dpp3* knock-out (ko) male mice (4 mice/genotype); Western blot analysis of selected upregulated proteins and gene expression analysis of genes potentially involved in the identified pathways.

**Results:**

We found that Dpp3 deficiency was associated with a proteomic signature in the bone matrix consistent with sustained oxidative stress and altered matrix turnover and pointing to a metabolic adaptation within the skeletal tissue. Accordingly, lack of Dpp3 resulted in a shift in lipid composition in the bone matrix, with enrichment of specific structural lipids and lack of others for energy production.

**Conclusion:**

Our work confirms the importance of DPP3 in the context of bone homeostasis and sheds some light on the matrix composition of DPP3-depleted bone. Owing to the foreseen translational implications of this evidence, further investigation is deserved to complete a comprehensive characterization.

**Supplementary Information:**

The online version contains supplementary material available at https://doi.org/10.1007/s40618-026-02949-6.

## Introduction

Peptidases represent a large family of proteins present in all living organisms, which catalyze the hydrolysis of peptide bonds either within the polypeptide chain (endopeptidases) or at the terminal ends (exopeptidases) in a broad range of biological substrates. Peptidases are involved in several pathophysiological processes by contributing to the degradation of off-function proteins and regulating biological processes crucial for cell homeostasis [1].

In particular, the dipeptidyl peptidases (DPPs) are ubiquitous enzymes that cleave dipeptides from the N-terminus of polypeptides. While sharing a common enzymatic mechanism, DPPs have different substrate specificities and structures [2]. Among them, DPP3 is a zinc-dependent exopeptidase targeting proline-containing peptides. It plays a key role in the regulation of blood pressure through the hydrolysis of the bioactive peptide angiotensin 2, and in pain modulation by targeting enkephalins and endorphins. It is also involved in inflammation and immune cell functions, and in carcinogenesis, by promoting cell proliferation, migration, survival, and metabolic adaptation. Moreover, DPP3 activates the antioxidant pathway Keap1-Nrf2 by promoting the release and nuclear translocation of Nrf2, which results in the expression of detoxifying genes; however, this mechanism does not appear to rely on DPP3 enzymatic activity [3].

In the bone, DPP3 has emerged as an important molecule for bone tissue homeostasis [4]. Indeed, it is expressed by skeletal cells, and we previously reported that genetic inactivation in the *Dpp3* knock-out (*Dpp3* ko) mouse model causes prolonged oxidative stress and an inflammatory microenvironment in the bone tissue that favors osteoclast formation and resorption function, with consequent bone loss. We also observed increased osteoid thickness and altered bone biomechanical characteristics in *Dpp3* ko mice and altered gene expression during in vitro differentiation of osteogenic lineage precursors [4]. These data suggested that in the absence of DPP3 a broad impairment of skeletal cell function occurs, involving both the osteoclast and the osteoblast lineage. Besides (or secondary to) derangements in bone remodeling, we also suspected altered composition and properties of the bone matrix in *Dpp3* ko mice. Indeed, we previously applied a non-linear optical imaging approach on undecalcified tissue sections of the lumbar spine of *Dpp3* ko and wild type (WT) mice and, taking advantage of the second harmonic generation signal produced by collagen fibers and of the high-energy vibrational response of lipids and proteins at specific wavelengths, we showed higher collagen and lipid content and altered collagen fiber orientation in the bone of *Dpp3* ko mice compared to controls. These features were accompanied by increased collagen secretion by *Dpp3* ko primary osteoblasts in vitro and abnormal expression of genes related to lipid transport and metabolism [5].

Bone is a composite material comprising organic (mainly collagen and non-collagenous proteins) and inorganic (mineral, water) components [6]. Its overall make-up is influenced by a plethora of local and systemic factors [7–9], and closely related to tissue function and biomechanical properties, i.e., to bone health [10]. Of note, lipids in bone have recently gained very much attention owing to their diverse functions and relevance to bone homeostasis [11]. Not only are lipids present in the bone marrow, but also in the mineralized hard tissue itself, as observed already 50 years ago [12]. In the mineralized matrix, lipids are tightly associated with proteins and minerals, and of course are important components of skeletal cells’ plasma membrane [11]. They influence bone cell survival and functions, bone mineralization, and critical signaling pathways, such as Wnt signaling [13]; in turn, their composition is modulated by pathophysiological conditions, such as age and hormonal status [14, 15], and environmental factors, such as diet [16]. Oxidative stress is strictly related to lipid metabolism: indeed, accumulation of reactive oxygen species damages macromolecules also including lipids, and in turn lipid peroxidation fuels oxidative stress [17]. Of note, the Nrf2 signaling pathway is a key regulator of lipid peroxidation, and accordingly we previously documented marked accumulation of lipid peroxidation adducts in the bone of *Dpp3* ko mice by 4-Hydroxynonenal (4-HNE) staining [4].

Altogether these data supported the hypothesis that bone matrix composition might change whether DPP3 is expressed or not. Here we exploited the *Dpp3* ko mouse model to go into greater detail of bone composition in the absence of DPP3, by performing proteomic and lipidomic analysis of bone in the *Dpp3* ko mouse compared to WT.

## Methods

### Animals

All the procedures involving mice were performed in accordance with ethical rules of the Institutional Animal Care and Use Committee of Humanitas Clinical and Research Center and with international laws (Italian Ministry of Health, protocol n.555/2023-PR). Animals were group-housed in a conventional animal facility, under a 12-h dark/light cycle, with water and food provided ad libitum. Specifically, mice were fed with an autoclavable certified complete universal vegetable diet (cat. n. DS811910G10R, Special Diet Services, Rosenberg, Germany), containing 19.1% proteins, 4.8% lipids, 3.9% fibers, Ca 1.01%, P 0.64%, Na 0.32%, Vit A 38580 IU/kg, Vit D3 1500 IU/kg, Vit E 105 IU/kg, Cu 12 mg/kg, Fe 150 mg/kg, Mn 99 mg/kg, Zn 120 mg/kg, Se 0.18 mg/kg. The generation of global *Dpp3* ko mice has been previously described [4]. For proteomic and lipidomic analysis, 6-month-old male C57BL/6 J WT and *Dpp3* ko mice (n = 4 per genotype) were asphyxiated under CO_2_, then the hindlimbs were excised, cleaned from soft tissue, and further processed as described below.

### Proteomics analysis

After carefully removing all the soft tissue around the hindlimbs of WT and *Dpp3* ko mice (6-month-old male mice, n = 4 per genotype), bone marrow (BM) cells were collected from the long bones by cutting the bone extremities, inserting the needle (21 G) of a syringe containing PBS/2% FBS/5 mM EDTA through the cut extremities into the medullary cavity and forcing the buffer through the cavity repeatedly, until the bone turned completely white. Then, total protein extracts were prepared from the femur and tibia cortex (no marrow) of a single limb of each mouse through a TRIzol-based method, as described [18]. For each sample, 15 mg total proteins underwent tryptic digestion (0.05 μg/μL trypsin in 50 mM ammonium bicarbonate at 37 °C overnight). High resolution mass spectrometry (HRMS) analysis was conducted using a Dionex Ultimate 3000 nano-LC system (Sunnyvale, CA, USA) connected to an Orbitrap Fusion™ Tribrid™ Mass Spectrometer (Thermo Scientific, Bremen, Germany) equipped with a nano electrospray ion source. Peptide mixtures were pre-concentrated onto a PepMap 100 C18 column (Thermo Scientific) and separated on EASY-Spray column ES902, packed with Thermo Scientific Acclaim PepMap RSLC C18, using mobile phase A (0.1% formic acid in water) and mobile phase B (0.1% formic acid in acetonitrile 20/80, v/v) at a flow rate of 0.300 μL/min and temperature set to 35 °C. The samples were injected in triplicate (5 μL each). MS spectra were collected over an m/z range of 375–1500 Da at 120,000 resolutions, operating in the data dependent mode TOP10 acquisition.

### Proteomics data processing and statistical analysis

All data processing and statistical analyses were performed in R using the Quantitative Proteomics Made Simple (QProMS) app (https://bioserver.ieo.it/shiny/app/qproms). Protein abundance data were obtained from Proteome Discoverer 2.5, and the “Abundances (Normalized)” values were used as input for downstream analyses. No additional normalization steps were applied. Prior to statistical analysis, proteins were filtered to retain only those identified in at least 75% of samples within each experimental group (n = 4 per group; KO vs WT). Additionally, proteins supported by fewer than two unique peptides were excluded to ensure robust protein identification. Missing values were imputed using a mixed imputation strategy implemented in the QProMS app, which integrates multiple approaches depending on the underlying missingness mechanism. Differential protein abundance between KO and WT groups was assessed using Welch’s t-test to account for unequal variances. Proteins were considered significantly differentially abundant if they met both a fold change threshold ≥ 1 and an adjusted p-value (Benjamini–Hochberg correction) ≤ 0.05.

### Lipidomics analysis

Flushed bone (femur and tibia, devoid of marrow elements) of 6-month-old male WT and *Dpp3* ko mice (n = 4 per genotype) was pulverized in a mortar with liquid N_2_. For lipid extraction, 200 μL H_2_O (LC–MS grade), 5 μL internal standard (SPLASH™ LIPIDOMIX™ Internal Standards, Avanti Polar, Sigma-Aldrich) and 1 mL dichloromethan:methanol (2:1, v:v) were added to each sample. Afterwards, samples were put in an ultrasound bath for 30 min, then centrifuged at 13,000 rpm for 30 min at 15 °C. The organic phase was collected and dried under N_2_ flow, and the pellet was solubilized in 100 μL of a solution containing 2-propanol:acetonitrile (90:10, v:v), 0.1% formic acid and 10 mM ammonium acetate. In parallel, a “Blank” sample (without internal standard) and a “Pool” sample (made of 10 μL of each reconstituted sample) were prepared as controls.

HRMS analysis was conducted using an Agilent 1290 Infinity II LC instrument (Agilent) connected to a ZenoTOF 7600 System (SCIEX) equipped with Turbo V™ Ion Source with ESI Probe. Chromatographic separation was achieved on a Kinetex® EVO C18 column using mobile phase A (ammonium acetate 10 mM in water:acetonitrile (60:40, v:v), 0.1% formic acid) and mobile phase B (ammonium acetate 10 mM in 2-propanol:acetonitrile (90:10, v:v), 0.1% formic acid) at a flow rate of 0.400 mL/min. The column and autosampler temperatures were set at 45 °C and 15 °C respectively. The sample injection volume was 5 μL; each sample was injected in duplicate. MS spectra were collected over an m/z range of 140–1500 Da, operating in the Information Dependent Acquisition (IDA®) mode. The data were elaborated using the MS-DIAL software v5.1 and the integrated reference database LipidBlast v68 and normalized on the weight of the powdered bone sample.

### Western blot protein analysis

Twenty micrograms (20 μg) of protein extracts prepared as described [18] were separated on a 12% SDS-PAGE, transferred to a nitrocellulose membrane, and probed with the following primary anti-mouse antibodies: Collagenase 3 (Mmp13) (orb238934, Biorbyt LLC, Durham, NC, USA; rabbit polyclonal antibody, validated on mouse kidney and lung and on the Jurkat and PC3 cell lines), neutrophil Elastase (ab310335, Abcam, Cambridge, UK; rabbit monoclonal antibody, validated on human and rat bone marrow tissue lysates) and CD36 (ab252922, Abcam; rabbit monoclonal antibody, validated on murine white and brown fat and spleen lysates), all diluted 1:1000 in 5% milk in 20 mM Tris-buffered saline, pH 7.6, 0.1% Tween 20 (TBST); Tuba1a (T5168, Sigma-Aldrich; mouse monoclonal antibody, validated on lysates of several cell lines including HeLa, Jurkat, Cos7, NIH-3T3, PC-12, RAT2, CHO, MDBK, and MDCK) diluted 1:5000 in 5% milk in TBST; and with an antibody for α-Actin (A2066, Sigma-Aldrich; rabbit polyclonal antibody, validated in the same cell lysates as above) diluted 1:2000 in 5% milk in TBST, then washed and probed with a secondary anti-rat antibody conjugated with horseradish peroxidase (HRP) and developed using the Clarity™ Western ECL kit (Bio-Rad).

Images were captured using the ChemiDoc™ MP Imaging System equipped with Image Lab™ Touch Software v2.0 (Bio-Rad) and analyzed using Fiji ImageJ 2.3.0/1.53f51; Java 1.8.0_172.

### Gene expression analysis

Total RNA was extracted from whole tissues using the TRIzol reagent (Qiagen), following the manufacturer’s instructions. Reverse transcription was carried out using 0.5 to 1.0 μg total RNA and High Capacity cDNA Reverse Transcription Kit (Applied Biosystems, Foster City, CA, USA). qPCR was performed using SsoAdvanced™ SYBR® Green Supermix (Bio-Rad) and gene-specific primers (available upon request). The amplification was performed using the ViiA7 Real-Time PCR Detection System (Applied Biosystems) with the following cycling conditions: cDNA denaturation and polymerase activation step at 95 °C for 20 s, 40 cycles of denaturation at 95 °C for 1 s and annealing at 60 °C for 20 s; extension step for 60 cycles at 65 °C for 30 s and melting curve analysis step at 65 °C to 95 °C with 0.5 °C increment for 2 s/step. The relative gene expression analysis of target genes was conducted following the comparative 2^−ΔCt^ method, and the normalized expression was calculated as arbitrary units (AU).

### Statistics

No statistical method was used to predetermine sample size. Data are presented as mean ± standard error of the mean (SEM). Comparison between groups was performed using Mann–Whitney test. Proteomics data were analyzed using the R-based algorithm Quantitative Proteomics Made Simple (QProMS) (https://bioserver.ieo.it/shiny/app/qproms); Principal Component Analysis (PCA) was performed to identify groupings within the whole dataset and differential expression analysis to determine which proteins are differently expressed in *Dpp3* ko vs WT bone. Functional interaction analysis of the differentially expressed proteins was performed using the STRING database v.12.0 [19]. Enrichment analysis was performed with EnrichR (https://maayanlab.cloud/Enrichr/) using Gene Ontology (GO) databases for biological process and molecular function enrichment. Pathway Enrichment Analysis (KEGG Pathways) was performed using STRING v12.0.

Lipidomics data were analyzed using the web-based platform MetaboAnalyst v6.0, with Pareto scaling for data normalization and following a univariate analysis method providing fold change, t-test, and Volcano plot. Differentially up- or down-regulated lipids between the two genotypes were defined as lipid changes with absolute values of fold change > 1.5 and p value < 0.05. Partial Least Squares Discriminant Analysis (PLS-DA) was conducted to achieve dimensionality reduction. Pathway Enrichment Analysis was performed using the free tool LIpid Pathway Enrichment Analysis (https://hyperlipea.org).

Statistical significance was considered when p < 0.05. Analyses were made with GraphPad Prism 10.0 (GraphPad Software, Inc., La Jolla, CA, USA).

Correlation analysis between the level of selected proteins and those of the lipid species with significantly different abundance in the flushed bone of *Dpp3* ko vs WT mice was conducted using the freely available webtool Social Science Statistics (socscistatistics.com) and verified with stata v18.

## Results

### Absence of DPP3 is associated with altered protein composition of the bone

**Fig. 1:**
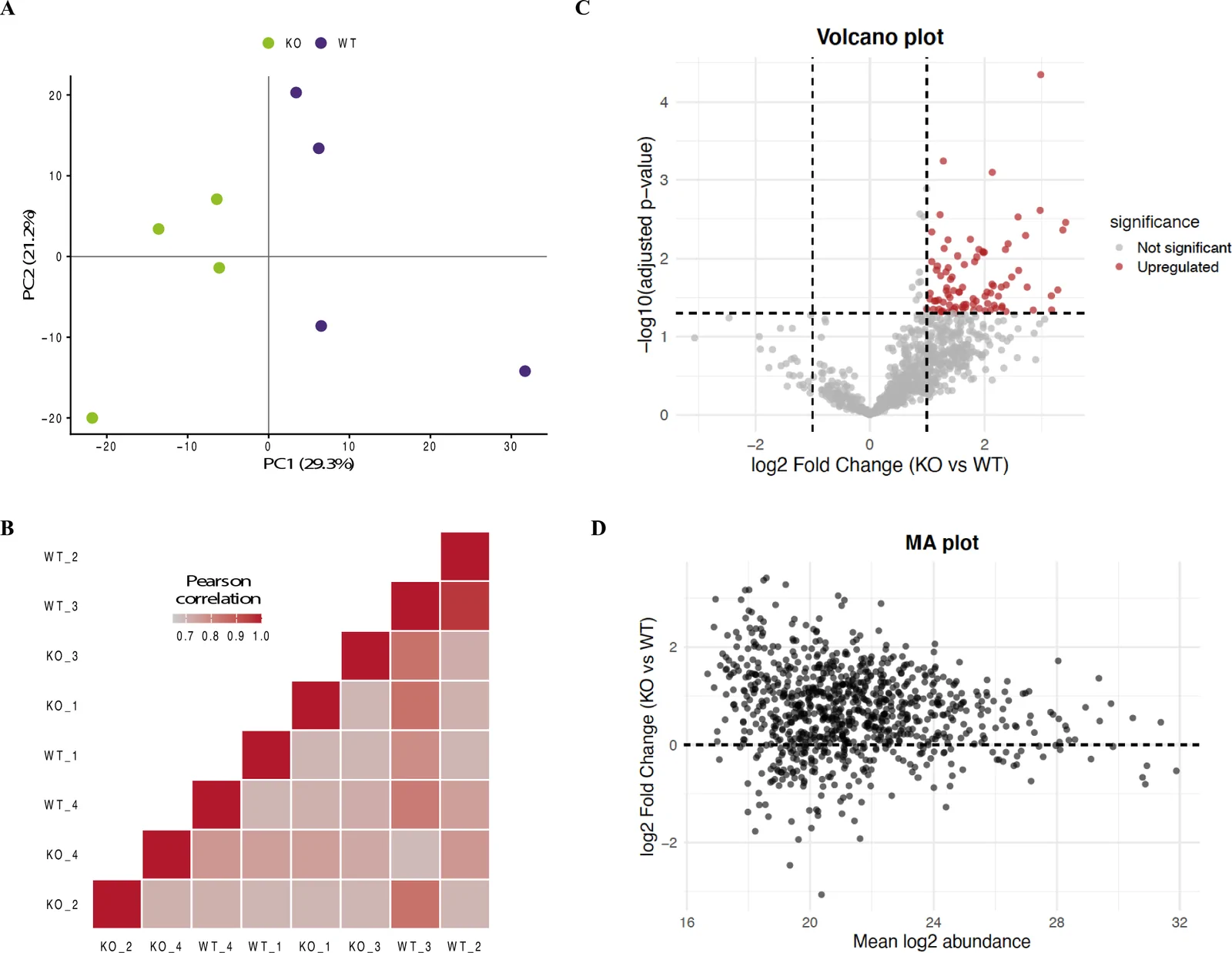
Proteomics analysis of the bone in *Dpp3* ko vs WT mice. (**A**) Principal Component Analysis (PCA) on the experimental samples (flushed bone of 6-month-old WT and *Dpp3* ko male mice, n = 4 per genotype). The x-axis (PC1) and y-axis (PC2) represent the first two principal components, which explain the largest variance in the data. (**B**) Correlation heatmap illustrating the relationships between all samples included in the experiment; pairwise correlation coefficients are displayed. High correlation values indicate similar proteomic profiles, suggesting consistency between replicates or related samples, while lower correlation values may point to variations or outliers. (**C**) Volcano plot visualizing the results of the differential expression analysis conducted on the proteins in the experimental samples. Each point represents a protein, with the x-axis displaying the difference (log2 fold change) in expression between conditions and the y-axis showing the negative log10 of the p-value. (**D**) MA plot, where M is the logged fold change between the abundance values and A is the average of the logged abundance, which can be used to determine whether the intensity differences between a particular microarray and a reference microarray (e.g., the artificial “average array”) depend on the magnitude of the intensity values. The plot shows symmetric distribution of log2 fold changes centered around zero, ruling out global bias towards upregulation

HRMS identified a total of 919 proteins in the bone of 6-month-old male *Dpp3* ko and WT mice. Principal component analysis (PCA) demonstrated a clear separation between *Dpp3* ko and WT samples, indicating distinct proteomic profiles (Fig. 1A). Correlation analysis further confirmed high consistency among biological replicates within each genotype (Fig. 1B). Differential expression analysis revealed 89 proteins with significantly increased abundance in *Dpp3* ko bone compared to WT (Fig. 1C; the full list of significantly differentially expressed proteins -DEPs- is reported in Supplementary Table 1). No proteins were found to be significantly downregulated, likely due to analytical limitations of the adopted data acquisition mode that impedes detection of small fold changes particularly in the case of low-abundance proteins. Importantly, the MA plot (Fig. 1D) showed a symmetric distribution of log2 fold changes centered around zero, indicating no global bias towards upregulation. Notably, several proteins displayed negative fold changes, confirming the presence of downregulation. However, these proteins were predominantly found at lower abundance levels, where higher variability and missingness are expected in DDA-based proteomics, thereby reducing statistical power to detect significant downregulation.

**Fig. 2:**
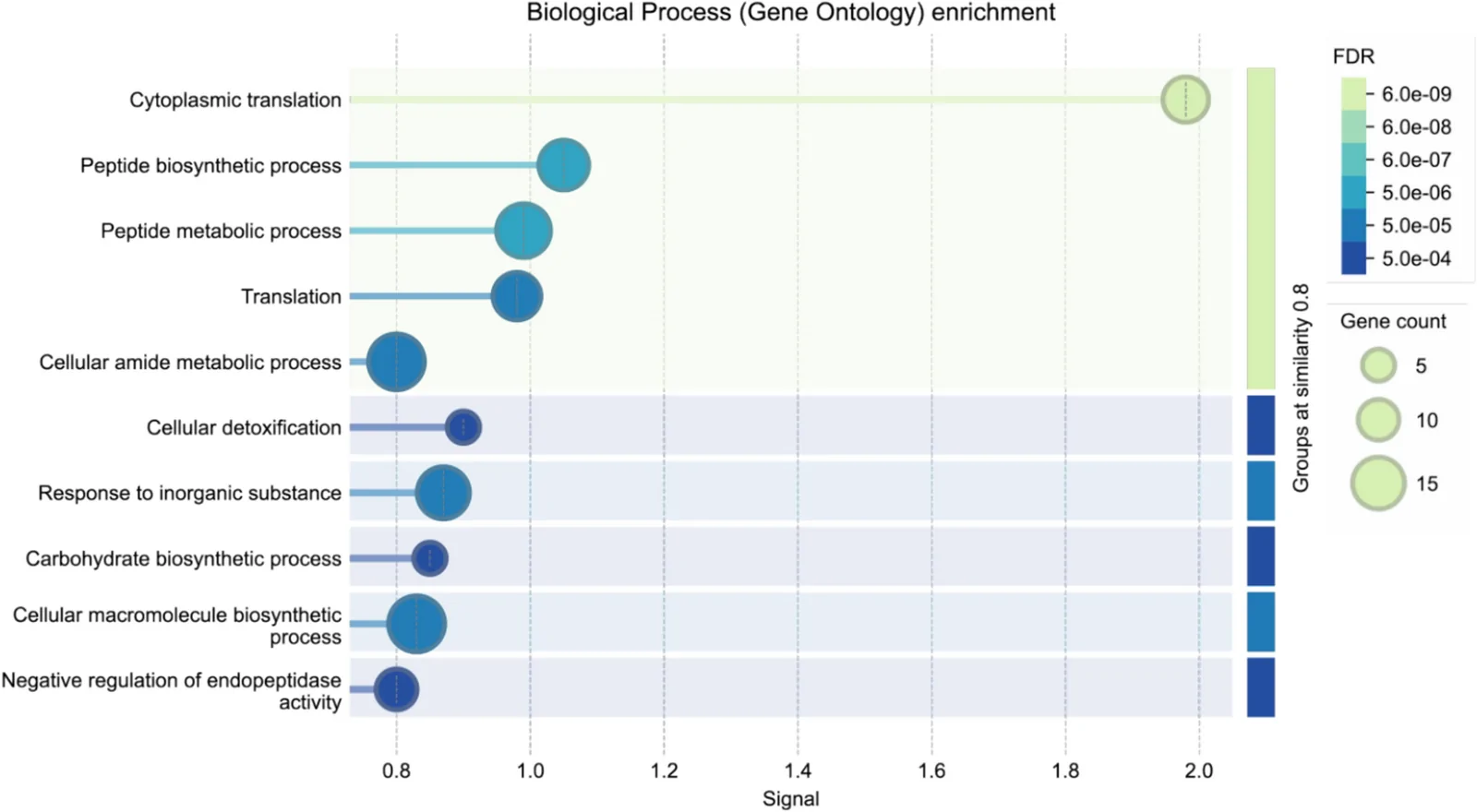
Gene Ontology enrichment analysis of the 89 differentially expressed proteins in the bone in *Dpp3* ko vs WT mice

Gene Ontology (GO) enrichment analysis delineated the functional implications of the set of DEPs. For what pertains to biological processes, GO analysis found clear enrichment in terms related to cytoplasmic translation, detoxification of reactive oxygen species, glycolysis and gluconeogenesis, long chain fatty acid transport, matrix remodeling, and regulation of organelle organization (Fig. 2).

GO Pathway analysis and Molecular function analysis revealed enrichment in several metabolic pathways including glycolysis, gluconeogenesis, carbon metabolism, arginine, and proline metabolism (Table 1), for what pertains to function, enrichment in the term Fatty Acid (FA) binding and transport (among others; Table 2).

**Table 1:** GO Pathway enrichment analysis (KEGG Pathways) in our dataset using STRING v12.0

#term ID	Term description	Count in network	FDR	Matching proteins
mmu03010	Ribosome	11 of 126	3.02e-09	Rps11, Rpl6, Rps4x, Rpl4, Rpl10a, Rps2, Rpl11, Rps16, Rps9, Rpl12, Rpl8
mmu04145	Phagosome	8 of 162	7.60e-05	Atp6v1b2, Atp6v1e1, Mpo, Thbs4, Sec22b, Tuba1a, Cd36, H2-D1
mmu00010	Glycolysis/Gluconeogenesis	5 of 65	0.0011	Aldoc, Pgam2, Akr1a1, Gpi1, Eno3
mmu01100	Metabolic pathways	18 of 1534	0.0036	Ckb, Atp6v1b2, P4ha1, Aldoc, Atp6v1e1, Pgam2, Akr1a1, Gsr, Pnp, Eprs, Gpi1, Impa1, Acadm, Gpx1, Ganab, Mdh1, Eno3, Cndp2
mmu01200	Carbon metabolism	5 of 122	0.0094	Aldoc, Pgam2, Gpi1, Mdh1, Eno3
mmu04657	IL-17 signaling pathway	4 of 89	0.0207	Mmp13, Mmp9, Hsp90b1, Hsp90aa1
mmu03050	Proteasome	3 of 46	0.0345	Psma7, Psmb2, Psmc3
mmu00330	Arginine and proline metabolism	3 of 52	0.0439	Ckb, P4ha1, Cndp2

Based on this and on prior knowledge in the literature, among the 89 DEPs in our dataset we selected four potentially interesting candidates for validation through Western blot analysis: Collagenase 3, alias Matrix metalloproteinase-13, Mmp-13; neutrophil elastase (Elane); CD36, also called Platelet Glycoprotein 4, Fatty Acid Translocase or Thrombospondin Receptor, owing to the diverse localization and functions of this protein; and Tubulin Alpha 1a (Tuba1a). Briefly, Collagenase 3 is an endopeptidase proteolytically cleaving collagens and a variety of extracellular matrix proteins [20]. It plays a key role in the process of bone formation and remodeling, as documented in mice and men [21–23]. It has been recently reported to be overexpressed in the cortical bone of db/db mice [24], after PTH stimulation [25] and upon enhanced oxidative stress in diverse pathophysiological contexts, including osteoarthritis [26, 27]. Neutrophil elastase is a proteoglycan-degrading enzyme that has been associated with inflammatory arthritis and osteoarthritis through activation of the pro-enzyme pro-MMP-13 [28], and with osteonecrosis of the femoral head, together with Mpo, ApoE, and Mmp9 [29], which in turn are also upregulated in the bone of *Dpp3* ko mice (Supplementary Table 1). CD36 acts as a regulator of osteoblast matrix mineralization, indeed Cd36 genetic deficiency causes low bone mass due to reduced osteoblast numbers and bone formation rate [30]; on the other hand, inhibition of the PPAR-g signaling pathway, including *Adipoq*, *Cd36* and *Fabp4*, was found to promote matrix mineralization in murine calvaria primary osteoblast cultures [31]. Together with CD47, CD36 mediates the stimulatory effect of thrombospondin 1 on osteoclast formation and resorbing function [32]. CD36 is also a scavenger receptor for oxidized low-density lipoproteins, anionic phospholipids, and long-chain fatty acids, and by associating with LRP5 and Frizzled leads to downregulation of the WNT signaling pathway [33]. Finally, Tuba1a is a major component of microtubules and is important for cytoskeletal organization. It has been found significantly upregulated in MC3T3-E1 preosteoblastic cells treated with a high dose of dexamethasone and proposed to play a role in glucocorticoids-induced reduction of osteoblastogenesis [34]. Moreover, Tuba1a expression level is associated with post-translational modifications of O-GlcNAc glycosylation, and based on public databases and bioinformatics analysis, it has been very recently attributed prognostic significance for human osteosarcoma, together with other glycosylation-related genes [35].

Overall, we thought that upregulation of Collagenase 3, Elane, CD36 and Tuba1a fit well with the low bone mass, increased oxidative stress and inflammation described in *Dpp3* ko mice [4].

**Fig. 3:**
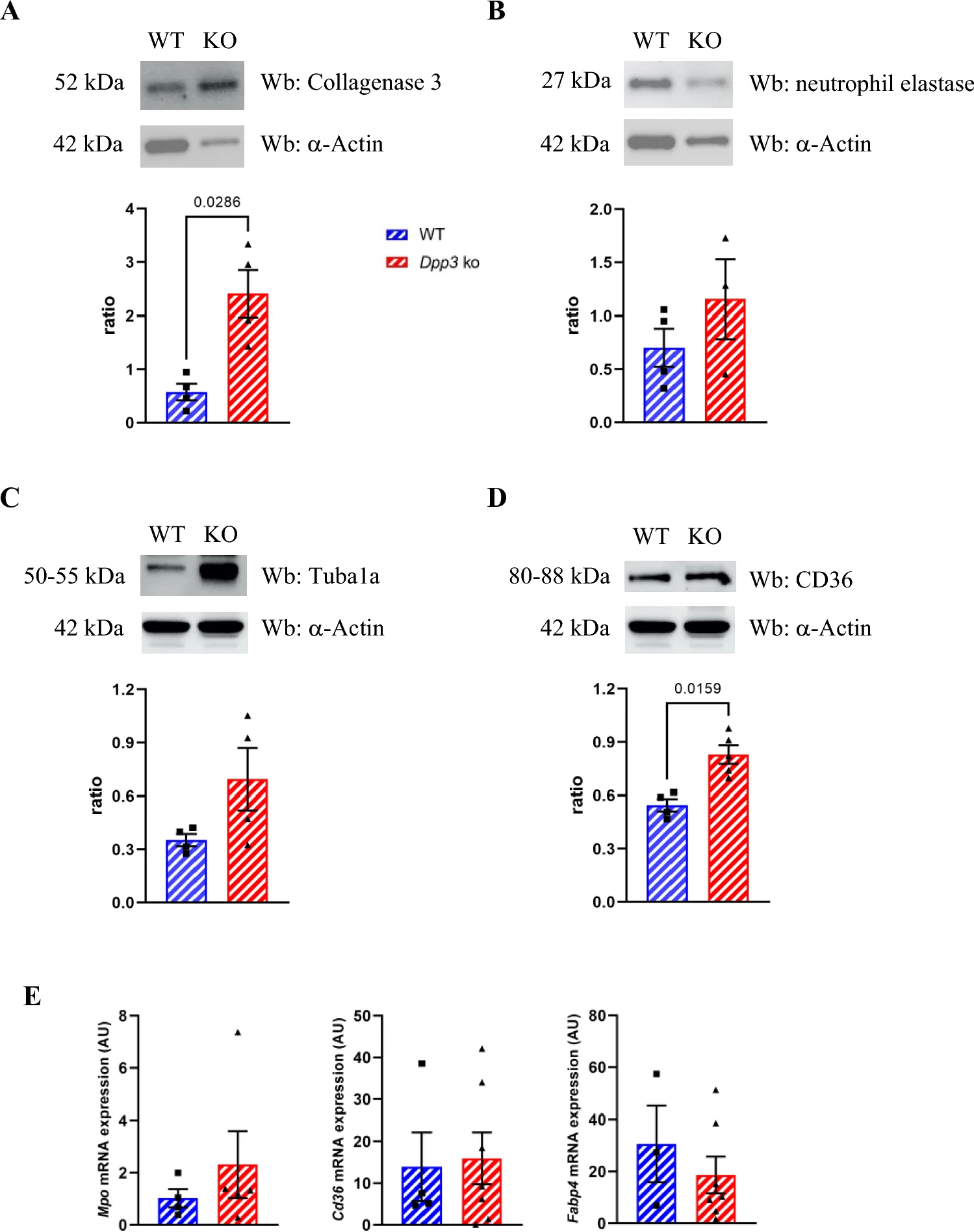
Western blot analysis of the total protein extracts from the flushed bone of 6-month-old WT and Dpp3 ko male mice (used for proteomics analysis) with (**A**) anti-Collagenase 3, (**B**) anti-Elastase, (**C**) anti-Tuba1a and (**D**) anti-CD36 antibodies; loading control: anti-α-Actin antibody. For each gel, one representative of two is reported. In each panel, the bar plot shows the relative density for each antibody’s pair, as determined using the ImageJ software; data are shown as mean and SEM; n = 3–5/genotype for each protein. Statistical analysis: Mann Whitney test. (**E**) RT-qPCR analysis of the indicated genes in the flushed bone of 6-month-old WT and *Dpp3* ko male mice (n = 4–7)

Indeed, Western Blot analysis confirmed that all these proteins were more abundant in *Dpp3* ko compared to WT flushed bone (Fig. 3A-D), and the upregulation was statistically significant for Collagenase 3 and CD36 (Fig. 3A and D).

Finally, for other candidate proteins (Mpo, CD36 and Fabp4) in our dataset we assessed the expression at the gene level in the flushed bone: *Mpo* had a trend in line with evidence at the protein level (i.e., higher in the mutant), while no difference was observed for *Cd36* and *Fabp4* (Fig. 3E), possibly suggesting that post-transcriptional and/or post-translational regulatory mechanisms described in other tissues may come into action here, too, and contribute to determining protein abundance [36, 37].

### Absence of DPP3 is associated with altered lipid composition of the bone

Lipidomics analysis of bone from the same animals included in the proteomics analysis identified in total 1140 lipids. Among them, 858 (75.3%) were even-chain lipids and the ratio between the relative amount of even- and odd-chain lipids was comparable in *Dpp3* ko and WT mice.

**Fig. 4:**
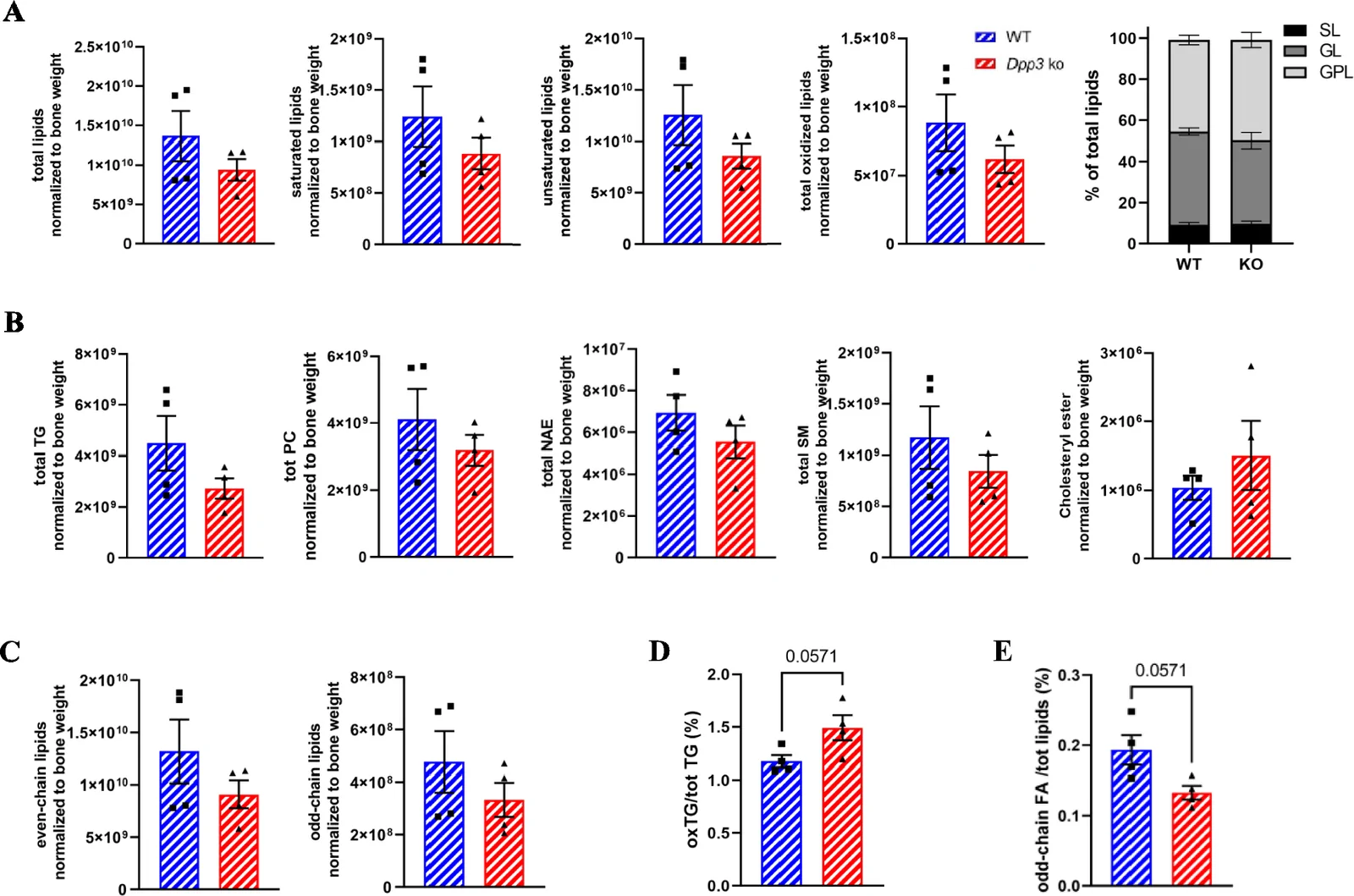
Descriptive analysis of lipids identified by lipidomics analysis in the experimental samples (flushed bone of 6-month-old WT and *Dpp3* ko male mice, n = 4 per genotype). (**A**) Relative amount of total lipids, saturated, unsaturated, and oxidized lipids, and percentages of the main categories. (**B**) Relative amount of total triglycerides (TG), phosphocolines (PC), N-Acylethanolamines (NAE), sphingomyelins (SM) and cholesteryl esters. (**C**) Relative amount of even-chain and odd-chain lipids. (**D**) Percentage of oxidized TG (oxTG) over total TG and (**E**) odd-chain FA over total odd-chain lipids. Data are presented as mean and SEM; Mann–Whitney test

Overall, 22 lipid classes were represented in our dataset (Supplementary Table 2). We observed a trend to lower total lipids and lower saturated, unsaturated, and oxidized lipids in the *Dpp3* ko vs WT bone, however significant difference was not reached and the relative abundance of the main lipid categories, comprising glycerolipids, glycerophospholipids and sphingolipids, was comparable in the two genotypes (Fig. 4A). This was not in contradiction with our previous report of higher lipid content in *Dpp3* ko bone [5], because in that case we applied NLO to spine sections and we cannot exclude that lipid composition differs depending on the skeletal site.

A trend to reduction was present also in individual lipid subclasses and more clearly in TG (Fig. 4B), while cholesteryl esters were slightly more abundant in *Dpp3* ko vs WT bone. Both even-chain and odd-chain lipids appeared slightly fewer in *Dpp3* ko compared to WT bone (Fig. 4C); at the same time, even-chain lipids were much more abundant than odd-chain in both genotypes and in each subfraction the composition resembled what was observed for the total lipids (not shown). Of note, the fraction of oxidized TG over total TG was higher in the *Dpp3* ko vs WT bone (Fig. 4D) and significantly strongly positively associated with the levels of CD36 and with Fabp4 (Supplementary Table 3), while the fraction of odd-chain FA over total odd-chain lipids was lower in the *Dpp3* ko vs WT bone (Fig. 4E) and significantly strongly negatively associated with the levels of CD36 (Supplementary Table 3). This could suggest that in the bone of *Dpp3* ko mice increased lipid uptake and oxidation occur owing to higher CD36 and Fabp4 expression.

The ratio of polyunsaturated vs saturated FA (PUFA/SFA) and the ratio of PUFA vs monounsaturated FA (PUFA/MUFA) did not significantly change in *Dpp3* ko compared to WT mice; the same was found for the ratio between palmitic acid, a 16-carbon long-chain FA (LCFA) highly relevant to bone pathophysiology [38], and its monounsaturated counterpart, palmitoleic acid (data not shown). These data could suggest preserved desaturase activity in *Dpp3* ko bone.

**Fig. 5:**
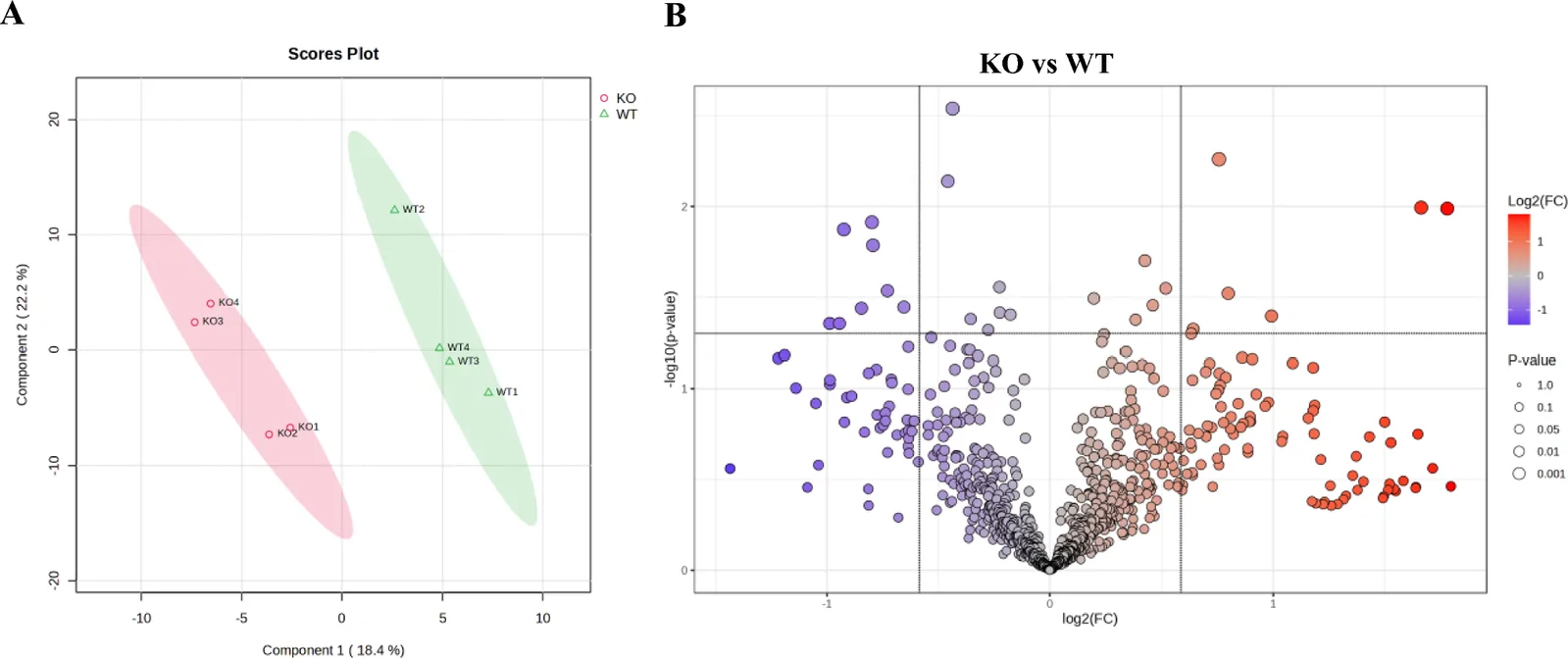
(**A**) PLS-DA of the lipid species identified in the experimental samples. (**B**) Volcano plot visualizing the results of the differential expression analysis conducted on the lipid species identified in the experimental samples

Partial least squares discriminant analysis (PLS-DA) on even-chain lipids highlighted a net separation between samples from animals of different genotype (Fig. 5A), illustrated also by the Volcano plot (Fig. 5B).

In particular, the differential analysis identified 9 lipids (mostly glycerolipids) with significantly lower abundance and 8 lipids (mostly glycerophospholipids) with significantly greater abundance in the flushed bone of *Dpp3* ko as compared to WT mice (Table 3). This evidence might suggest a shift in lipid composition, whereby absence of DPP3 seemed to result in relative enrichment of structural lipids (glycerophospholipids) and decrease of others (glycerolipids) useful for energy production.

**Table 2:** Top 10 significant p-values and q-values for the analysis of the 89 upregulated proteins in our dataset with GO Molecular Function 2025

term	p-value	q-value	overlap_genes
mRNA Binding (GO:0003729)	0.000500	0.048397	[DDX3Y, DDX5, SSB, CAPRIN1, HNRNPU, RPS2, EIF3A]
rRNA Binding (GO:0019843)	0.000955	0.048397	[RPS9, RPL12, RPL11]
ATPase Activity, Coupled to Transmembrane Movement of Ions, Rotational Mechanism (GO:0044769)	0.001283	0.048397	[ATP6V1B2, ATP6V1E1]
Proton-Transporting ATPase Activity, Rotational Mechanism (GO:0046961)	0.001283	0.048397	[ATP6V1B2, ATP6V1E1]
Long-Chain Fatty Acid Transmembrane Transporter Activity (GO:0005324)	0.001512	0.048397	[FABP4, CD36]
Fatty Acid Transmembrane Transporter Activity (GO:0015245)	0.002024	0.053975	[FABP4, CD36]
Protein Homodimerization Activity (GO:0042803)	0.003505	0.080109	[HSP90AA1, SFPQ, SNX2, EPRS1, IMPA1, SLC4A1, ACTN4, APOE, B2M]
ATPase Binding (GO:0051117)	0.004536	0.081695	[ATP1B3, ATP6V1E1, TOR1AIP1]
MHC Class II Protein Complex Binding (GO:0023026)	0.005182	0.081695	[HSP90AA1, B2M]
Low-Density Lipoprotein Particle Receptor Binding (GO:0050750)	0.005182	0.081695	[APOE, HSP90B1]

Pathway enrichment analysis generated by the LIpid Pathway Enrichment Analysis (LIPEA) free tool identified several pathways, and among them the glycerophospholipid metabolism and choline metabolism in cancer was statistically significant after Bonferroni correction (Table 4).

**Table 3:** Lipids with significantly different relative abundance in the bone of *Dpp3* ko as compared to WT mice

Lipid	Category	FC	Log2 (FC)	raw p value
TG 60:1|TG 16:0_26:0_18:1	Glycerolipids	0.53944	− 0.89047	0.003881
TG 62:1|TG 16:0_28:0_18:1	Glycerolipids	0.58892	− 0.76385	0.005334
TG 64:1|TG 16:0_16:0_32:1	Glycerolipids	0.61567	− 0.69978	0.01653
TG 54:1|TG 18:0_18:0_18:1	Glycerolipids	0.56951	− 0.8122	0.023364
Cer 46:1;O3|Cer 20:1;O2/26:0;O	Sphingolipids	0.57468	− 0.79916	0.024064
TG 32:0|TG 8:0_12:0_12:0	Glycerolipids	0.64437	− 0.63404	0.026957
TG 56:1|TG 16:0_24:0_16:1	Glycerolipids	0.5194	− 0.94509	0.028729
TG 64:2|TG 16:0_16:1_32:1	Glycerolipids	0.5345	− 0.90373	0.031447
MG 36:0	Glycerolipids	0.63413	− 0.65715	0.032059
HBMP 60:9|HBMP 22:4/18:1_20:4	Glycerophospholipids	1.6901	0.7571	0.007129
PC 34:5|PC 16:2_18:3	Glycerophospholipids	3.2965	1.7209	0.011909
Hex2Cer 42:2;O2|Hex2Cer 18:1;O2/24:1	Sphingolipids	1.5532	0.63523	0.016629
PI 40:7	Glycerophospholipids	3.5212	1.8161	0.024071
TG 52:6;O|TG 16:1_18:2_18:3;O	Glycerolipids	1.6037	0.68144	0.03778
PC 38:4|PC 18:0_20:4	Glycerophospholipids	2.0491	1.035	0.041601
PC 22:0|PC 11:0_11:0	Glycerophospholipids	1.9315	0.9497	0.043344
LPC 22:0	Glycerophospholipids	1.7695	0.82337	0.04508

We performed correlation analysis between each single lipid species and the 4 DEPs in our dataset associated with lipid transport and metabolism (i.e., CD36, Fabp4, Acadm and ApoE) (Supplementary Table 4). We found that CD36 was strongly negatively correlated with the less abundant and positively correlated with the more abundant lipid species, but never reached significance. Correlations with Fabp4 had essentially the same trend, and in particular TG 54:1|TG 18:0_18:0_18:1, TG 32:0|TG 8:0_12:0_12:0, and TG 56:1|TG 16:0_24:0_16:1 were significantly strongly negatively correlated with Fabp4 levels. Acadm was significantly strongly correlated with all the less abundant lipids, and the same for ApoE with most of them. Overall, this would agree with the hypothesis of increased FA breakdown and lower energy stores in the bone of *Dpp3* ko mice.

**Fig. 6:**
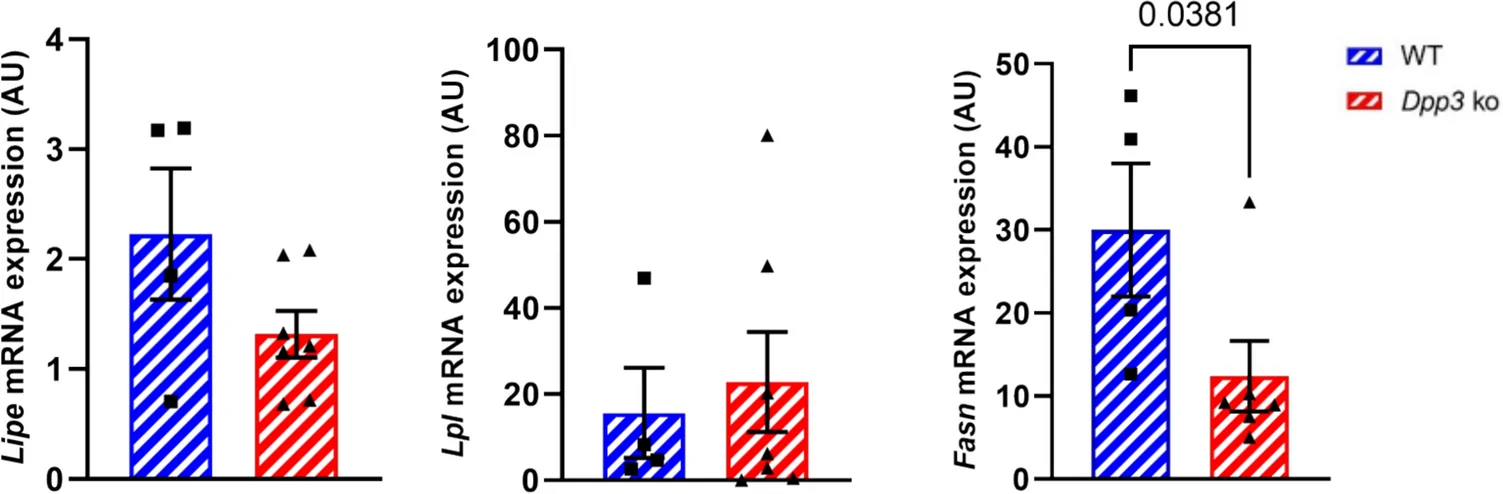
RT-qPCR analysis of genes involved in FA metabolism, in the flushed bone of 6-month-old WT and *Dpp3* ko male mice (n = 4–7). All data are presented as mean and SEM. Mann Whitney test

On the other hand, we performed expression analysis of genes encoding enzymes crucial for lipid metabolism (as well as for bone metabolism and regeneration, [39]), namely the hormone-sensitive lipase E (*Lipe*), important for hydrolysis of TG to FFA; the lipoprotein lipase (*Lpl*), marker of lipolysis; and FA synthase (*Fasn*), marker of de novo lipogenesis. We found a trend to lower expression of *Lipe*, no difference in the expression of *Lpl,* and significantly lower expression of *Fasn* in the flushed bone of *Dpp3* ko as compared to WT mice (Fig. 6).

**Fig. 7:**
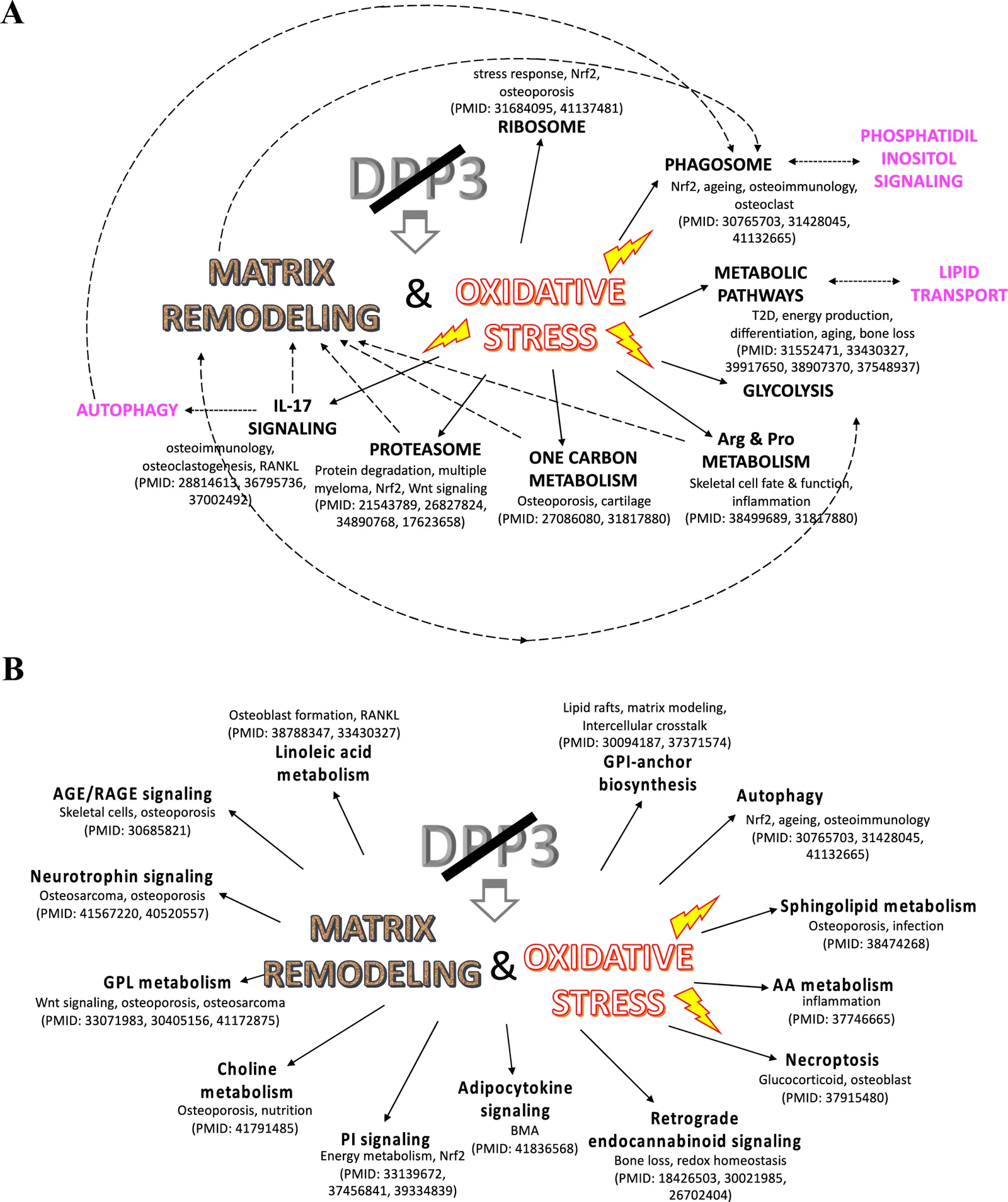
Schematic representation of the interrogation of the list of terms (in bold case) identified by Pathway Enrichment Analysis of the differentially expressed proteins (**panel A**) and of the lipid species with significantly different abundance in the flushed bone of *Dpp3* ko vs WT mice (**panel B**), with the terms “oxidative stress” and “matrix remodeling”. For each pair of terms, representative relevant papers (by means of PMIDs) and related keywords are provided. Arg: Arginine; Pro: Proline; PI: Phosphatidyl Inositol; AA: Arachidonic Acid; GPI: GlycosilPhosphatidylInositol

Finally, identifying oxidative stress and matrix remodeling as the core features of the bone in the absence of DPP3, we used them to interrogate the list of terms identified in the Pathway enrichment analyses (Fig. 7). This in silico research highlighted a functional connection between the two datasets and allowed better delineating the overall picture, giving hints for future validation studies.

## Discussion

In the present work we strengthen the relationship between DPP3 and bone biology by providing insights into bone tissue composition in the absence of DPP3. We conducted proteomic and lipidomic analysis of flushed bone in 6-month-old WT and *Dpp3* ko male mice and found that the proteomic signature of Dpp3-deficient bone was consistent with sustained oxidative stress and altered matrix turnover and pointed to a metabolic adaptation within the skeletal tissue.

We selected four candidate DEPs for validation and confirmed statistically significant upregulation of two of them (Collagenase 3 and CD36). In addition, works published by our own and other groups support the significance of several other DEPs of our dataset in our model and pave the way to future mechanistic studies.

For example, we uncovered a higher abundance of proteins involved in detoxification of reactive oxygen species (e.g., Mpo, Elane, Gsr, Gpx) and other related mechanisms, which agreed with our previous report of higher inflammation and oxidative stress in the bone and expansion of the neutrophil population (which were more activated, too) in the bone marrow of *Dpp3* ko mice [4]. We speculate that enrichment in ribosomal proteins may derive from enhanced protein turnover (accordingly, some proteasomal subunits were upregulated in the *Dpp3* ko bone; see Table 4) and from oxidative stress-induced ribosomal damage and ribosomal stress leading to accumulation of ribosome-free ribosomal proteins [40, 41]. The higher expression of Impa1 protein (Importin alpha 1), a nuclear transport receptor belonging to karyopherins family, involved also in the assembly of RNA stress granules with a pro-survival role [42], fits in the overall scenario of increased oxidative stress in *Dpp3* ko bone. At the same time, increased Impa1 might favor bone matrix remodeling: indeed, karyopherins have been implicated in the pathological remodeling of the extracellular matrix of the shoulder tendon through the nucleocytoplasmic transport of key mediators, including HIF-1α, TGF-β, and MMP-9 [43]; we might speculate a similar mechanism can be executed in the bone. Accordingly, indeed, proteins involved in matrix remodeling (e.g., Collagenase 3, Mmp9) were upregulated in *Dpp3* ko bone compared to WT in our dataset, also in line with impaired bone remodeling and collagen content [4, 5].

**Table 4:** Pathway enrichment analysis (generated using LIPEA tool) of the list of lipid species with significantly different abundance in the bone of *Dpp3* ko as compared to WT mice

Pathway name	Pathway lipids	p-value	Benjamini correction	Bonferroni correction
Glycosylphosphatidylinositol (GPI)-anchor biosynthesis	3	0.038956663	0.079461058	0.779133256
Inositol phosphate metabolism	9	0.112982432	0.14122804	1
Linoleic acid metabolism	25	0.286972363	0.302076172	1
Sphingolipid metabolism	21	0.02757922	0.079461058	0.551584409
Sphingolipid signaling pathway	9	0.005101917	0.034012777	0.102038331
Glycerophospholipid metabolism	26	0.000142174	0.002843484	0.002843484
Arachidonic acid metabolism	75	0.65633052	0.65633052	1
Alpha-Linolenic acid metabolism	23	0.26696853	0.2966317	1
Autophagy—animal	4	0.051649688	0.79461058	1
Autophagy—other	3	0.038956663	0.079461058	0.779133256
Necroptosis	4	0.051649688	0.079461058	1
Phosphatidylinositol signaling system	11	0.136545434	0.160641687	1
Retrograde endocannabinoid signaling	8	0.100995708	0.134660944	1
Neurotrophin signaling pathway	3	0.038956663	0.079461058	0.779133256
AGE-RAGE signaling pathway in diabetic complications	2	0.026118298	0.079461058	0.522365952
Adipocytokine signaling pathway	3	0.038956663	0.079461058	0.779133256
Insulin resistance	4	0.051649688	0.079461058	1
Leishmaniasis	4	0.051649688	0.079461058	1
Tuberculosis	5	0.064198747	0.091712496	1
Choline metabolism in cancer	5	0.001453452	0.014534522	0.029069045

Another protein in our dataset with a potentially related function is Immt (inner membrane mitochondrial protein, *alias* Mitofilin), key component of the mitochondrial contact site and cristae junction organizing system. Mitofilin is critical for maintaining mitochondrial membrane structure and function [44]. Its overexpression in rat cortical neurons suppressed mitochondrial fission and increased mitochondrial length in neurites [45]. Mitochondrial dynamics are of course crucial also for skeletal cell fate and function and dependent on nutrient availability. For example, in osteogenic cells simultaneous glucose and palmitate supplementation induced mitochondrial fission [46], while glucose restriction resulted in increased mitochondrial fusion and elongation [47]. Moreover, lack of Mitofilin led to senescent bone marrow mesenchymal stromal cells and bone loss [48]. Therefore, the interplay between Dpp3 and Mitofilin in these cellular mechanisms would be worth further investigation.

Enrichment in proteins related to glucose and lipid metabolism (e.g., the glycolytic enzyme Aldolase C, Aldoc; the medium-chain acyl-CoA dehydrogenase, Acadm; the lipid chaperone Fabp4; the lipid scavenger Cd36, and ApoE) suggested that lack of DPP3 might impact on energy metabolism in bone. Accordingly, the amount of oxTG was higher in *Dpp3* ko bone, accompanied by lower expression of *Lipe*, playing an important role in the hydrolysis of TG stored in lipid droplets, and of *Fasn*, key enzyme in LCFA de novo biosynthesis. Interestingly, lipid-related genes involved in the β-oxidation pathway (such as *Acadm*) have recently found up-regulated in the flushed bone of aged (16-month-old) C57BL/6 J male mice, together with upregulated oxidative stress genes and downregulated osteoblast-related genes, as compared to young (2-month-old) mice [49].

Regarding lipids, in general in bone most of them are in the marrow, and a minority in the mineralized tissue, either associated with cellular structures or as part of the extracellular matrix itself, and locally produced or derived from the blood circulation (indeed, dietary fatty acids can be incorporated into bone). Lipids in bone exert diverse functions [11]: phospholipids and cholesterol have a structural role in the plasma membrane of all cells in the bone tissue, in matrix vesicles and in the bone mineralized extracellular matrix; triglycerides are the main form of energy storage and important nutrients together with cholesterol, and a mean of intercellular crosstalk and intracellular signaling together with phospholipids [11]. Sphingomyelin is essential in bone formation, but an excess has been associated with bone resorption; ceramides have a time- and dose-dependent effect in the osteoblast lineage and a pro-apoptotic function at high concentration; elevated levels have been also positively associated with senescence and with bone resorption markers; S1P is important for hematopoietic and mesenchymal skeletal cell homing, osteoblast-osteoclast crosstalk, and osteoclast differentiation [50]. Carnitines/acylcarnitines are essential in the context of fatty acid oxidation and contribute to energy metabolism, mitochondrial homeostasis, epigenetic regulation, endocrine regulation, inflammation and immune homeostasis, and signal transduction. Recently, they have been shown to enhance Nrf2 signaling by preventing Nrf2-Keap1 binding and reducing its degradation, hence a decrease in carnitine levels results in overactivation of osteoclasts, ultimately leading to osteoporosis [51]. FA are a very important source of energy both for osteoblast maturation and mineralized matrix synthesis [52], and for osteoclast differentiation and resorption function [53]. FFA are lipid-modifiers of Wnt proteins which require this post-translation modification to be secreted [11]. Moreover, palmitic acid has concentration-dependent effect on osteoblast function [46] and increases while palmitoleic acid inhibits RANKL-induced osteoclastogenesis [54, 55]. UFA have a protective effect on bone by suppressing osteoclastogenesis [56] and PUFA are the substrates for the local production of prostaglandins, which impact skeletal cells in several ways [57].

In this study, we found no gross alterations, but mild changes of lipid composition of the mineralized bone in the absence of DPP3, including, most notably, lower total TG, possibly resulting in impaired bone metabolism, and higher cholesteryl esters, possibly owing to higher uptake by osteoblasts from low-density lipoproteins by means of scavenger receptors class B. On the other hand, PLS-DA of even-chain lipids clearly separated samples of the two genotype groups and differential analysis identified a set of lipids with significantly different abundance in the *Dpp3* ko compared to WT bone: namely, 8 glycerolipids were decreased (and only 2 increased) and 6 glycerophospholipids increased in the *Dpp3* ko. Of course, these findings require confirmation through validation of specific targets, nonetheless they raise the hypothesis of changes impacting the overall energetic status and membrane remodeling in the absence of DPP3. In our interpretation, in the *Dpp3* ko bone skeletal cells have preserved or increased lipid handling, while lipid neo-synthesis is defective. We cannot define a causal relation between the two, but we think that a consequent metabolic impairment can be reasonably expected, also in line with the reported osteogenic impairment [4]. Moreover, diverse pieces of evidence might point to intense membrane remodeling, in line with higher osteoclast numbers in the *Dpp3* ko bone, release of proteases and possibly also extracellular vesicles with a very varied cargo [58]. This speculation is supported by the results of pathway enrichment analysis on our proteomics and lipidomics datasets shown in Tables 1, 2, 3 and 4 and in Fig. 7. We envisage that clearer insights and confirmation of this hypothesis might be obtained by performing metabolomics analysis in distinct cell populations.

Our work has some limitations: the first is related to the sample size, which is low, thus limits the robustness of our findings and imposes caution in interpretation. Also, the limited number of samples likely allowed us to unveil only strong effects of the lack of DPP3, while we cannot exclude that milder, but biologically relevant changes have been missed in our analysis. Moreover, our study was conceived as exploratory, so no statistical method was used to predetermine sample size, which was set to 4 mice per genotype (while it was higher for the evaluation of gene expression levels). This number was acceptable for our purpose and also if compared to published work [59]. Of note, the results obtained appeared to fit well with previous data: indeed, interrogating the terms of Pathway enrichment analysis of proteomics and lipidomics data with the terms “oxidative stress” and “bone remodeling” (which synthesize the bone phenotype of *Dpp3* ko mice, as described [4]) highlighted clear connections between them. Therefore, despite the limited sample size, this work is a continuation and a step forward from our original publication, and better delineate the overall picture of the skeletal phenotype in relation to absence of DPP3.

Furthermore, systemic parameters such as serum lipids and glucose, that based on prior knowledge [3, 4] could be expected to change in the context of Dpp3 deficiency and then in turn to impact on bone homeostasis, were not measured here. We also acknowledge that the diet composition influences bone metabolism and composition (in mouse models, and even more importantly in humans), therefore it is important to place our results in the specific environmental conditions in which they have been obtained. Finally, some interesting aspects have not been addressed in this work, but might be considered for future developments of this work: for example, it would be interesting to prospectively analyze the proteomic and lipidomic profile drawn here both at a younger and at an older age than that of mice in this study, to define the dynamics of these changes; to include also female mice, in order to investigate the interplay between sex hormones and DPP3 expression in this context; and to complement this work with other omics analyses, in order to establish the underlying molecular mechanism. Moreover, it would be interesting to also analyze the inorganic component of the bone matrix, to follow up the initial observation of lower mineralization rate in the *Dpp3* ko compared to WT [4] and clarify whether it’s not only a matter of mineral quantity but also of quality.

Despite these open questions, which make the comprehensive characterization of Dpp3-deficient bone a future goal, our work confirms the importance of DPP3 with respect to bone homeostasis, whereby lack of DPP3 results in sustained oxidative stress and unbalanced osteoblast/osteoclast function, with consequent changes in bone tissue protein and lipid composition which feed into each other in a condition that we expect to worsen overtime. Further investigation might be deserved in the framework of pathological conditions affecting the skeleton.

## Supplementary Information

Below is the link to the electronic supplementary material.

Supplementary file1 (DOCX 79 KB)
