# Supplementary material for: Multiomics profiling of bone in the Dpp3-deficient mice unveils altered protein and lipid composition
Source: J Endocrinol Invest. 2026 Aug 3;49(10):2897–912. doi: 10.1007/s40618-026-02949-6 (PMC13627175; doi:10.1007/s40618-026-02949-6)
Supplement: Supplementary file 1 [file 40618_2026_2949_MOESM1_ESM.docx]

**Multiomics profiling of bone in the *Dpp3-*deficient mice unveils altered protein and lipid composition**

Emilia Bevacqua^1^, Federica Violi^2^, Valentina Granata^2^, Fabio Bedin^3^, Mariarosaria Santillo^4^, Ciro Menale^4^, Gherardo Mazziotti^5,6^, Cristina Sobacchi^1,7,*^

**Supplementary Information**

**Supplementary Table 1**. Complete list of the significantly upregulated proteins in Dpp3-deficient bone; the proteins analyzed by Western blot are highlighted in green. Blue boundaries indicate proteins included in the correlation analysis (see Supplementary Tables 2 and 3).

| **Accession** | **Description** | **Coverage**  **[%]** | **# Peptides** | **# PSMs** | **# Unique Peptides** | **# Razor Peptides** | **log2 mean intensity KO_1** | **log2 mean intensity KO_2** | **log2 mean intensity KO_3** | **log2 mean intensity KO_4** | **log2 mean intensity WT_1** | **log2 mean intensity WT_2** | **log2 mean intensity WT_3** | **log2 mean intensity WT_4** | **KO_vs_WT**  **log10(p.val)** | **KO_vs_WT Fold Change** |
| --- | --- | --- | --- | --- | --- | --- | --- | --- | --- | --- | --- | --- | --- | --- | --- | --- |
| **O08547** | Vesicle-trafficking protein SEC22b | 14 | 2 | 3 | 2 | 0 | 18.31 | 17.875 | 18.776 | 18.733 | 15.389 | 15.092 | 15.371 | 15.911 | 4.349 | 2.983 |
| **P01899** | H-2 class I histocompatibility antigen, D-B alpha chain | 34 | 10 | 61 | 8 | 2 | 23.275 | 22.856 | 23.508 | 23.1 | 22.021 | 21.998 | 21.507 | 22.102 | 3.243 | 1.278 |
| **P50518** | V-type proton ATPase subunit E 1 | 21 | 4 | 23 | 4 | 0 | 20.994 | 21.768 | 21.748 | 21.019 | 19.704 | 18.724 | 18.888 | 19.673 | 3.098 | 2.135 |
| **Q61576** | Peptidyl-prolyl cis-trans isomerase FKBP10 | 14 | 5 | 6 | 5 | 0 | 18.541 | 18.804 | 19.079 | 20.567 | 15.29 | 16.86 | 16.3 | 16.652 | 2.611 | 2.972 |
| **Q60668** | Heterogeneous nuclear ribonucleoprotein D0 | 11 | 4 | 44 | 2 | 1 | 21.708 | 21.319 | 21.453 | 22.132 | 20.831 | 20.077 | 20.206 | 20.612 | 2.555 | 1.221 |
| **P25444** | Small ribosomal subunit protein uS5 | 18 | 5 | 28 | 5 | 0 | 21.145 | 21.544 | 21.514 | 22.173 | 20.191 | 18.684 | 18.47 | 18.686 | 2.526 | 2.586 |
| **Q02053** | Ubiquitin-like modifier-activating enzyme 1 | 10 | 7 | 15 | 7 | 0 | 19.705 | 20.541 | 19.573 | 21.359 | 16.899 | 17.64 | 15.27 | 17.69 | 2.456 | 3.42 |
| **Q8BHN3** | Neutral alpha-glucosidase AB | 6 | 4 | 8 | 4 | 0 | 20.387 | 19.625 | 18.999 | 21.711 | 16.368 | 17.595 | 17.264 | 16.009 | 2.359 | 3.371 |
| **Q9R0P5** | Destrin | 22 | 3 | 12 | 3 | 0 | 21.357 | 21.729 | 21.802 | 22.278 | 20.26 | 20.869 | 20.869 | 20.821 | 2.334 | 1.087 |
| **P32067** | Lupus La protein homolog | 10 | 3 | 6 | 3 | 0 | 19.953 | 18.253 | 19.477 | 20.171 | 16.866 | 16.622 | 16.421 | 17.069 | 2.289 | 2.719 |
| **P68510** | 14-3-3 protein eta | 33 | 9 | 70 | 4 | 1 | 23.287 | 22.53 | 23.37 | 22.172 | 21.322 | 21.799 | 20.432 | 20.8 | 2.24 | 1.752 |
| **P32261** | Antithrombin-III | 15 | 5 | 6 | 5 | 0 | 19.94 | 18.907 | 19.055 | 18.893 | 17.916 | 17.772 | 17.487 | 18.181 | 2.233 | 1.36 |
| **Q9CWK8** | Sorting nexin-2 | 5 | 2 | 2 | 2 | 0 | 17.603 | 18.942 | 17.044 | 18.75 | 16.27 | 15.508 | 16.159 | 14.755 | 2.183 | 2.412 |
| **P68369** | Tubulin alpha-1A chain [OS=Mus musculus] | 42 | 14 | 233 | 5 | 10 | 24.814 | 25.246 | 24.739 | 25.808 | 23.846 | 24.15 | 23.58 | 23.858 | 2.123 | 1.293 |
| **O08997** | Copper transport protein ATOX1 | 47 | 3 | 26 | 3 | 0 | 22.37 | 21.811 | 21.954 | 22.314 | 20.986 | 19.578 | 19.659 | 20.62 | 2.11 | 1.901 |
| **Q00898** | Alpha-1-antitrypsin 1-5 | 24 | 7 | 101 | 2 | 0 | 21.465 | 20.254 | 21.346 | 22.121 | 20.131 | 17.925 | 18.874 | 18.792 | 2.109 | 2.366 |
| **P07901** | Heat shock protein HSP 90-alpha | 25 | 15 | 61 | 6 | 0 | 20.442 | 21.389 | 20.494 | 21.843 | 18.257 | 19.929 | 18.69 | 19.421 | 2.082 | 1.968 |
| **Q9R1P3** | Proteasome subunit beta type-2 | 15 | 2 | 5 | 2 | 0 | 19.349 | 19.711 | 19.171 | 19.093 | 16.375 | 18.167 | 17.256 | 17.551 | 2.074 | 1.994 |
| **P53026** | Large ribosomal subunit protein uL1 | 23 | 6 | 19 | 6 | 0 | 21.163 | 21.061 | 21.367 | 22.389 | 20.566 | 19.616 | 18.701 | 19.187 | 2.07 | 1.978 |
| **P07759** | Serine protease inhibitor A3K | 26 | 8 | 83 | 4 | 4 | 23.059 | 23.493 | 22.382 | 23.336 | 21.548 | 21.839 | 22.1 | 20.667 | 2.031 | 1.529 |
| **Q61362** | Chitinase-3-like protein 1 | 12 | 3 | 5 | 3 | 0 | 17.792 | 17.371 | 18.338 | 18.872 | 16.344 | 16.292 | 16.112 | 16.172 | 2.017 | 1.863 |
| **Q9JII6** | Aldo-keto reductase family 1 member A1 | 13 | 3 | 10 | 3 | 0 | 20.403 | 20.52 | 20.022 | 21.071 | 19.428 | 19.517 | 19.203 | 19.524 | 1.962 | 1.086 |
| **Q61656** | Probable ATP-dependent RNA helicase DDX5 | 12 | 7 | 19 | 5 | 2 | 20.803 | 21.638 | 21.437 | 22.692 | 19.914 | 20.283 | 19.007 | 20.054 | 1.961 | 1.827 |
| **Q91WN1** | DnaJ homolog subfamily C member 9 | 14 | 2 | 5 | 2 | 0 | 18.666 | 17.253 | 18.707 | 18.74 | 17.291 | 16.221 | 16.495 | 16.776 | 1.923 | 1.646 |
| **P49710** | Hematopoietic lineage cell-specific protein | 22 | 9 | 68 | 9 | 0 | 22.523 | 23.037 | 23.062 | 22.995 | 22.211 | 21.297 | 21.263 | 22.135 | 1.905 | 1.178 |
| **Q9Z2U0** | Proteasome subunit alpha type-7 | 31 | 5 | 25 | 5 | 0 | 20.604 | 20.907 | 20.935 | 21.929 | 19.718 | 20.255 | 19.931 | 19.013 | 1.884 | 1.364 |
| **Q921T2** | Torsin-1A-interacting protein 1 | 8 | 3 | 5 | 3 | 0 | 20.372 | 20.441 | 20.84 | 20.478 | 19.895 | 19.411 | 19.502 | 18.662 | 1.853 | 1.165 |
| **P47791-2** | Isoform Cytoplasmic of Glutathione reductase, mitochondrial | 9 | 2 | 6 | 2 | 0 | 19.749 | 18.701 | 17.842 | 20.542 | 15.911 | 17.343 | 16.478 | 16.712 | 1.849 | 2.597 |
| **P11247** | Myeloperoxidase | 49 | 31 | 373 | 29 | 0 | 26.345 | 26.322 | 26.304 | 26.621 | 25.707 | 24.827 | 25.327 | 24.425 | 1.83 | 1.326 |
| **Q99KF1** | Transmembrane emp24 domain-containing protein 9 | 9 | 3 | 13 | 3 | 0 | 21.542 | 21.186 | 21.94 | 22.09 | 21.094 | 20.767 | 19.771 | 20.2 | 1.78 | 1.232 |
| **P14152** | Malate dehydrogenase, cytoplasmic | 31 | 8 | 58 | 8 | 0 | 23.086 | 23.459 | 22.253 | 23.238 | 21.932 | 22.349 | 21.116 | 20.91 | 1.765 | 1.432 |
| **Q9Z1T2** | Thrombospondin-4 | 7 | 5 | 28 | 3 | 0 | 19.989 | 20.524 | 21.864 | 20.254 | 18.996 | 19.371 | 16.878 | 17.48 | 1.765 | 2.476 |
| **Q3UP87** | Neutrophil elastase | 19 | 5 | 77 | 5 | 0 | 23.848 | 24.485 | 24.288 | 25.138 | 23.723 | 23.501 | 22.411 | 22.503 | 1.734 | 1.405 |
| **P62281** | Small ribosomal subunit protein uS17 | 30 | 4 | 5 | 4 | 0 | 19.674 | 20.341 | 20.479 | 21.616 | 19.927 | 18.275 | 17.467 | 17.916 | 1.673 | 2.131 |
| **P01872** | Immunoglobulin heavy constant mu | 7 | 2 | 8 | 2 | 0 | 18.296 | 20.189 | 19.83 | 19.422 | 17.066 | 17.503 | 15.38 | 18.274 | 1.663 | 2.379 |
| **P62702** | Small ribosomal subunit protein eS4 | 35 | 10 | 43 | 10 | 0 | 21.786 | 22.318 | 22.672 | 23.591 | 21.514 | 20.797 | 20.559 | 18.881 | 1.652 | 2.154 |
| **P62814** | V-type proton ATPase subunit B, brain isoform | 7 | 2 | 3 | 2 | 0 | 19.573 | 18.629 | 18.581 | 20.256 | 16.276 | 16.92 | 16.001 | 18.671 | 1.636 | 2.293 |
| **Q60715** | Prolyl 4-hydroxylase subunit alpha-1 | 12 | 4 | 11 | 4 | 0 | 17.99 | 19.051 | 19.242 | 21.264 | 16.692 | 17.004 | 16.796 | 16.07 | 1.635 | 2.746 |
| **P01887** | Beta-2-microglobulin | 16 | 2 | 21 | 2 | 0 | 22.352 | 22.49 | 22.375 | 22.523 | 21.118 | 21.761 | 20.227 | 20.176 | 1.633 | 1.614 |
| **O88685** | 26S proteasome regulatory subunit 6A | 6 | 2 | 2 | 2 | 0 | 18.944 | 18.484 | 19.85 | 19.939 | 17.536 | 18.475 | 18.161 | 17.695 | 1.628 | 1.338 |
| **P22599** | Alpha-1-antitrypsin 1-2 | 40 | 11 | 123 | 3 | 0 | 21.264 | 20.243 | 21.056 | 20.845 | 19.539 | 18.36 | 16.258 | 16.123 | 1.599 | 3.282 |
| **Q02013** | Aquaporin-1 | 11 | 2 | 18 | 2 | 0 | 20.044 | 21.074 | 21.802 | 20.935 | 19.905 | 20.092 | 19.278 | 19.257 | 1.591 | 1.33 |
| **P13020** | Gelsolin | 37 | 18 | 115 | 18 | 0 | 22.954 | 23.461 | 23.652 | 24.701 | 21.776 | 21.929 | 22.03 | 23.205 | 1.591 | 1.457 |
| **Q08857** | Platelet glycoprotein 4 | 7 | 2 | 3 | 2 | 0 | 18.732 | 18.482 | 17.447 | 17.609 | 17.107 | 16.291 | 15.442 | 17.223 | 1.572 | 1.552 |
| **Q04447** | Creatine kinase B-type | 38 | 10 | 89 | 10 | 0 | 23.25 | 23.14 | 22.673 | 24.943 | 22.19 | 22.227 | 20.122 | 21.299 | 1.571 | 2.042 |
| **P21550** | Beta-enolase | 42 | 15 | 240 | 12 | 0 | 24.897 | 25.292 | 24.699 | 24.38 | 23.251 | 24.46 | 22.726 | 22.598 | 1.57 | 1.558 |
| **Q8VDM6** | Heterogeneous nuclear ribonucleoprotein U-like protein 1 | 4 | 3 | 3 | 3 | 0 | 19.3 | 19.673 | 19.285 | 18.662 | 18.745 | 16.535 | 17.034 | 16.176 | 1.559 | 2.108 |
| **Q8VIJ6** | Splicing factor, proline- and glutamine-rich | 11 | 6 | 35 | 6 | 0 | 22.228 | 23.07 | 22.465 | 23.514 | 21.629 | 21.7 | 22.307 | 21.406 | 1.557 | 1.058 |
| **P11352** | Glutathione peroxidase 1 | 24 | 4 | 21 | 4 | 0 | 20.793 | 20.731 | 19.784 | 20.921 | 20.195 | 19.264 | 18.924 | 18.404 | 1.54 | 1.361 |
| **Q9D1M7** | Peptidyl-prolyl cis-trans isomerase FKBP11 | 9 | 2 | 6 | 2 | 0 | 17.983 | 20.934 | 17.882 | 21.132 | 15.269 | 15.475 | 17.612 | 16.899 | 1.524 | 3.169 |
| **Q62095** | ATP-dependent RNA helicase DDX3Y | 5 | 3 | 5 | 3 | 0 | 17.067 | 18.146 | 18.418 | 20.126 | 16.166 | 16.947 | 16.102 | 15.575 | 1.519 | 2.242 |
| **P23492** | Purine nucleoside phosphorylase | 21 | 3 | 11 | 3 | 0 | 18.431 | 20.535 | 20.271 | 20.628 | 17.948 | 17.986 | 17.953 | 17.942 | 1.519 | 2.009 |
| **P23116** | Eukaryotic translation initiation factor 3 subunit A | 2 | 2 | 4 | 2 | 0 | 17.953 | 19.086 | 18.134 | 19.614 | 16.711 | 17.818 | 16.943 | 17.744 | 1.502 | 1.393 |
| **P41245** | Matrix metalloproteinase-9 | 9 | 5 | 18 | 5 | 0 | 20.976 | 20.961 | 20.142 | 21.336 | 20.213 | 19.302 | 19.01 | 17.687 | 1.488 | 1.801 |
| **P07356** | Annexin A2 | 65 | 19 | 222 | 19 | 0 | 24.873 | 25.315 | 25.747 | 25.813 | 24.448 | 25.036 | 23.577 | 24.47 | 1.482 | 1.054 |
| **P97370** | Sodium/potassium-transporting ATPase subunit beta-3 | 8 | 2 | 3 | 2 | 0 | 19.529 | 18.62 | 19.224 | 20.278 | 18.871 | 17.707 | 18.009 | 18.28 | 1.471 | 1.196 |
| **Q9CQI6** | Coactosin-like protein | 56 | 7 | 42 | 7 | 0 | 21.953 | 22.429 | 21.501 | 22.38 | 21.416 | 21.322 | 19.927 | 20.973 | 1.459 | 1.156 |
| **P04117** | Fatty acid-binding protein, adipocyte | 45 | 6 | 34 | 6 | 0 | 21.463 | 22.695 | 21.86 | 22.27 | 20.439 | 21.144 | 20.423 | 21.749 | 1.456 | 1.133 |
| **Q8CAQ8** | MICOS complex subunit Mic60 | 15 | 8 | 28 | 8 | 0 | 21.28 | 22.221 | 22.728 | 22.306 | 20.205 | 21.617 | 18.922 | 20.381 | 1.456 | 1.852 |
| **P35979** | Large ribosomal subunit protein uL11 | 49 | 5 | 52 | 5 | 0 | 22.251 | 22.158 | 21.864 | 22.605 | 21.714 | 20.909 | 19.92 | 21.231 | 1.442 | 1.276 |
| **P05063** | Fructose-bisphosphate aldolase C | 14 | 3 | 25 | 1 | 0 | 18.366 | 18.621 | 20.044 | 20.024 | 17.5 | 18.799 | 16.124 | 16.413 | 1.425 | 2.055 |
| **P08113** | Endoplasmin | 25 | 15 | 92 | 15 | 0 | 22.844 | 23.587 | 23.27 | 24.741 | 21.744 | 22.019 | 20.442 | 23.023 | 1.417 | 1.804 |
| **P06745** | Glucose-6-phosphate isomerase | 23 | 11 | 81 | 11 | 0 | 23.07 | 23.622 | 23.189 | 23.58 | 22.155 | 22.481 | 20.249 | 21.873 | 1.414 | 1.675 |
| **P14131** | Small ribosomal subunit protein uS9 | 27 | 4 | 15 | 4 | 0 | 20.759 | 20.932 | 21.175 | 22.636 | 20.5 | 18.768 | 20.507 | 19.167 | 1.411 | 1.64 |
| **Q60605** | Myosin light polypeptide 6 | 56 | 9 | 117 | 6 | 1 | 25.047 | 24.516 | 25.14 | 25.329 | 24.288 | 24.231 | 22.467 | 23.496 | 1.406 | 1.387 |
| **P04919** | Band 3 anion transport protein | 11 | 8 | 19 | 8 | 0 | 19.713 | 21.444 | 19.593 | 21.344 | 18.68 | 20.035 | 17.416 | 17.291 | 1.402 | 2.168 |
| **Q6NZB0** | DnaJ homolog subfamily C member 8 | 15 | 2 | 5 | 2 | 0 | 19.658 | 18.095 | 18.314 | 18.967 | 18.056 | 15.514 | 14.99 | 17.245 | 1.391 | 2.307 |
| **P62918** | Large ribosomal subunit protein uL2 | 17 | 4 | 12 | 4 | 0 | 20.695 | 20.873 | 21.99 | 22.707 | 20.826 | 19.081 | 19.569 | 20.356 | 1.382 | 1.608 |
| **Q61166** | Microtubule-associated protein RP/EB family member 1 | 21 | 3 | 19 | 3 | 0 | 20.701 | 19.599 | 20.941 | 21.069 | 20.006 | 17.913 | 19.544 | 18.882 | 1.381 | 1.492 |
| **P47911** | Large ribosomal subunit protein eL6 | 26 | 7 | 33 | 7 | 0 | 21.16 | 22.682 | 22.746 | 23.606 | 20.928 | 21.006 | 19.895 | 21.708 | 1.377 | 1.664 |
| **Q6ZWN5** | Small ribosomal subunit protein uS4 | 27 | 7 | 30 | 7 | 0 | 21.571 | 22.363 | 22.709 | 23.541 | 21.713 | 21.189 | 18.59 | 19.48 | 1.371 | 2.303 |
| **P56399** | Ubiquitin carboxyl-terminal hydrolase 5 | 3 | 2 | 19 | 2 | 0 | 19.412 | 17.623 | 18.579 | 19.508 | 16.022 | 15.144 | 16.783 | 18.4 | 1.365 | 2.193 |
| **P45952** | Medium-chain specific acyl-CoA dehydrogenase, mitochondrial | 13 | 4 | 12 | 4 | 0 | 18.857 | 21.077 | 21.029 | 20.644 | 18.353 | 20.016 | 17.555 | 18.048 | 1.362 | 1.908 |
| **O55023** | Inositol monophosphatase 1 | 7 | 2 | 4 | 2 | 0 | 17.849 | 19.263 | 18.413 | 19.546 | 16.292 | 17.605 | 17.016 | 18.282 | 1.36 | 1.469 |
| **Q8CGC7** | Bifunctional glutamate/proline--tRNA ligase | 2 | 2 | 4 | 2 | 0 | 16.986 | 17.374 | 17.934 | 19.294 | 15.877 | 16.487 | 16.222 | 16.509 | 1.359 | 1.623 |
| **P08226** | Apolipoprotein E | 56 | 14 | 162 | 14 | 0 | 24.641 | 24.344 | 24.673 | 25.07 | 23.459 | 24.511 | 22.983 | 23.773 | 1.353 | 1.001 |
| **Q8VEK3** | Heterogeneous nuclear ribonucleoprotein U | 14 | 8 | 52 | 8 | 0 | 22.018 | 22.647 | 21.771 | 23.162 | 21.591 | 20.626 | 20.569 | 21.993 | 1.352 | 1.205 |
| **P97384** | Annexin A11 | 4 | 2 | 35 | 1 | 0 | 18.003 | 18.342 | 18.068 | 19.219 | 17.511 | 18.057 | 17.146 | 16.461 | 1.351 | 1.114 |
| **P33435** | Collagenase 3 | 17 | 5 | 14 | 5 | 0 | 18.265 | 17.618 | 20.935 | 21.611 | 15.748 | 17.689 | 14.608 | 17.686 | 1.343 | 3.174 |
| **P57780** | Alpha-actinin-4 | 33 | 23 | 205 | 9 | 0 | 20.42 | 23.299 | 24.548 | 21.546 | 19.972 | 19.459 | 20.293 | 18.683 | 1.341 | 2.852 |
| **P49290** | Eosinophil peroxidase | 25 | 15 | 61 | 13 | 0 | 21.849 | 23.01 | 21.908 | 23.054 | 22.13 | 20.685 | 19.467 | 19.119 | 1.341 | 2.105 |
| **Q9CXW4** | Large ribosomal subunit protein uL5 | 13 | 2 | 21 | 2 | 0 | 20.403 | 20.712 | 20.979 | 22.425 | 20.062 | 19.91 | 17.679 | 19.633 | 1.338 | 1.809 |
| **Q03734** | Serine protease inhibitor A3M | 16 | 5 | 49 | 1 | 0 | 19.746 | 18.769 | 19.178 | 16.806 | 17.111 | 16.541 | 16.552 | 16.256 | 1.331 | 2.01 |
| **Q61171** | Peroxiredoxin-2 | 36 | 7 | 41 | 7 | 0 | 22.434 | 23.216 | 23.182 | 23.584 | 21.753 | 21.566 | 20.547 | 22.829 | 1.329 | 1.43 |
| **Q9D1A2** | Cytosolic non-specific dipeptidase | 5 | 2 | 4 | 2 | 0 | 18.853 | 19.99 | 19.49 | 20.052 | 17.183 | 18.363 | 19.07 | 18.689 | 1.327 | 1.27 |
| **Q9D8E6** | Large ribosomal subunit protein uL4 | 23 | 7 | 38 | 7 | 0 | 21.101 | 21.748 | 21.447 | 22.907 | 21.416 | 20.31 | 20.06 | 20.435 | 1.325 | 1.246 |
| **Q60865** | Caprin-1 | 5 | 2 | 15 | 2 | 0 | 20.704 | 17.495 | 20.652 | 19.509 | 17.646 | 17.067 | 17.291 | 16.862 | 1.323 | 2.374 |
| **Q6WVG3** | BTB/POZ domain-containing protein KCTD12 | 27 | 6 | 30 | 6 | 0 | 21.163 | 21.612 | 20.747 | 22.266 | 20.552 | 21.045 | 19.183 | 19.527 | 1.318 | 1.37 |
| **Q8BJS4** | SUN domain-containing protein 2 | 20 | 11 | 31 | 11 | 0 | 20.822 | 21.564 | 21.628 | 22.779 | 20.303 | 20.157 | 20.591 | 20.785 | 1.316 | 1.239 |
| **O70250** | Phosphoglycerate mutase 2 | 50 | 11 | 155 | 9 | 2 | 25.141 | 24.882 | 24.602 | 23.996 | 23.695 | 24.209 | 22.777 | 21.952 | 1.303 | 1.497 |

PSM: Peptide-Spectrum Match

| **Lipid categories** | **Subclasses** |
| --- | --- |
| Fatty Acyls [FA] | free fatty acids [FFA] |
|  | N-acyl ethanolamines [NAE] |
|  | acylcarnitine [CAR] |
| Sterol lipids | cholesteryl esters [CE] |
| Glycerolipids [GL] | monoacylglycerols [MG] |
|  | diacylglycerols [DG] |
|  | triacylglycerols [TG] |
| Glycerophospholipids [GPL] | phosphatidylglycerol [PG] |
|  | phosphatidylethanolamines [PE] |
|  | phosphatidylcholines [PC] |
|  | phosphatidylinositol [PI] |
|  | phosphatidylserine [PS] |
|  | lysophosphatidylethanolamines [LPE] |
|  | lysophosphatidylcholines [LPC] |
|  | ether-linked phospholipids [EtherPE, EtherPC, EtherLPE, EtherLPC, EtherPG] |
|  | cardiolipin [CL] |
|  | N-acyl-lysophosphatidylethanolamine [LNAPE] |
|  | hemibismonoacylglycerophosphate [HBMP] |
| Sphingolipids [SL] | sphingomyelins [SM] |
|  | ceramides [CER] |
|  | hexosylceramides [HexCER] |
| Prenol lipids | coenzyme Q |

**Supplementary Table 2**. Lipid categories and classes represented in our dataset of lipids identified through lipidomics analysis of flushed bone of 6-month-old WT and *Dpp3* ko male mice (n=4 per genotype)

**Supplementary Table 3.** Correlation analysis between the level of selected differentially expressed proteins and lipid classes with different abundance in the flushed bone of *Dpp3* ko vs WT mice (n=4 mice per genotype). Red color indicates statistically significant correlation (p < 0.05).

|  | **vs CD36** | | **vs Fabp4** | | **vs Acadm** | | **vs ApoE** | |
| --- | --- | --- | --- | --- | --- | --- | --- | --- |
| **Lipid class** | **Spearman’s**  **ρ** | **Two-tailed**  **p value** | **Spearman’s**  **ρ** | **Two-tailed**  **p value** | **Spearman’s**  **ρ** | **Two-tailed**  **p value** | **Spearman’s**  **ρ** | **Two-tailed**  **p value** |
| **OxTG/TG** | 0.8810 | 0.0039 | 0.8333 | 0.010 | 0.5238 | 0.183 | 0.4286 | 0.289 |
| **odd chain FA/tot lipids** | -0.7619 | 0.028 | -0.3571 | 0.385 | -0.4286 | 0.289 | -0.4762 | 0.233 |

**Supplementary Table 4.** Correlation analysis between the level of selected differentially expressed proteins and lipid species with significantly different abundance in the flushed bone of *Dpp3* ko vs WT mice (n=4 mice per genotype). Red color indicates statistically significant correlation (p < 0.05).

|  | **vs CD36** | | **vs Fabp4** | | **vs Acadm** | | **vs ApoE** | |
| --- | --- | --- | --- | --- | --- | --- | --- | --- |
| **Lipid** | **Spearman’s**  **ρ** | **Two-tailed**  **p value** | **Spearman’s**  **ρ** | **Two-tailed**  **p value** | **Spearman’s**  **ρ** | **Two-tailed**  **p value** | **Spearman’s**  **ρ** | **Two-tailed**  **p value** |
| **TG 60:1\|TG 16:0_26:0_18:1** | -0.6190 | 0.102 | -0.6905 | 0.058 | -0.9286 | < 0.001 | -0.7381 | 0.037 |
| **TG 62:1\|TG 16:0_28:0_18:1** | -0.4762 | 0.233 | -0.6667 | 0.071 | -0.9524 | < 0.001 | -0.7143 | 0.047 |
| **TG 64:1\|TG 16:0_16:0_32:1** | -0.4524 | 0.260 | -0.6667 | 0.071 | -0.9524 | < 0.001 | -0.6905 | 0.058 |
| **TG 54:1\|TG 18:0_18:0_18:1** | -0.5238 | 0.183 | -0.7381 | 0.037 | -0.9286 | < 0.001 | -0.8571 | 0.0065 |
| **Cer 46:1;O3\|Cer 20:1;O2/26:0;O** | -0.1190 | 0.779 | -0.2381 | 0.570 | -0.6905 | 0.058 | -0.3810 | 0.352 |
| **TG 32:0\|TG 8:0_12:0_12:0** | -0.5000 | 0.207 | -0.7143 | 0.047 | -0.9762 | < 0.001 | -0.6190 | 0.102 |
| **TG 56:1\|TG 16:0_24:0_16:1** | -0.5238 | 0.183 | -0.7381 | 0.037 | -0.9286 | < 0.001 | -0.8571 | 0.0065 |
| **TG 64:2\|TG 16:0_16:1_32:1** | -0.4048 | 0.320 | -0.6190 | 0.102 | -0.8571 | 0.0065 | -0.8810 | 0.0039 |
| **MG 36:0** | -0.4762 | 0.233 | -0.5476 | 0.160 | -0.9048 | 0.0020 | -0.5238 | 0.183 |
| **HBMP 60:9\|HBMP 22:4/18:1_20:4** | 0.4971 | 0.230 | 0.1317 | 0.756 | -0.2395 | 0.568 | 0.3593 | 0.382 |
| **PC 34:5\|PC 16:2_18:3** | 0.5714 | 0.139 | 0.2857 | 0.493 | 0.0000 | 1.000 | 0.4286 | 0.289 |
| **Hex2Cer 42:2;O2\|Hex2Cer 18:1;O2/24:1** | 0.2857 | 0.493 | 0.1667 | 0.693 | -0.3095 | 0.456 | 0.2619 | 0.531 |
| **PI 40:7** | 0.4762 | 0.233 | 0.4762 | 0.233 | 0.2619 | 0.531 | 0.6905 | 0.058 |
| **TG 52:6;O\|TG 16:1_18:2_18:3;O** | -0.1667 | 0.693 | -0.2143 | 0.610 | -0.6667 | 0.071 | -0.1190 | 0.779 |
| **PC 38:4\|PC 18:0_20:4** | -0.0238 | 0.955 | 0.0714 | 0.867 | -0.2143 | 0.610 | 0.0952 | 0.823 |
| **PC 22:0\|PC 11:0_11:0** | 0.4524 | 0.260 | 0.3333 | 0.420 | -0.1429 | 0.736 | 0.3571 | 0.385 |
| **LPC 22:0** | 0.4762 | 0.233 | 0.1905 | 0.651 | -0.1667 | 0.693 | 0.3810 | 0.352 |
